# Application potential of stem/progenitor cell-derived extracellular vesicles in renal diseases

**DOI:** 10.1186/s13287-018-1097-5

**Published:** 2019-01-08

**Authors:** Xiao Sun, Huanyu Meng, Wuqing Wan, Min Xie, Chuan Wen

**Affiliations:** Division of Hematology and Tumor, Children’s Medical Center, The Second Xiangya Hospital, Central South University, No.139,Renmin road, Changsha, Hunan People’s Republic of China

**Keywords:** Extracellular vesicle, Stem / progenitor cell, Renal disease

## Abstract

Extracellular vesicles (EVs) are nanometer-sized and membrane-bound vesicles, including exosomes and microvesicles. EVs can deliver bioactive macromolecules such as proteins, lipids, and nucleic acids, allowing intercellular communication in multicellular organisms. EVs are secreted by all cell types including stem/progenitor cells. Stem/progenitor cell-derived EVs have been identified to exert immunomodulatory effects on target cells through transferring protein molecules as well as regulatory effects on the phenotype of target cells through fusion with the target cells membrane and/or through direct endocytosis by target cells to transfer nucleic acid substances (such as mRNA, miRNA) to the target cells. In both human and animal models, the use of stem/progenitor cells (such as bone marrow mesenchymal stromal cells) has been shown to promote the recovery of kidney diseases such as acute kidney injury and chronic kidney disease. Stem/progenitor cell-derived extracellular vesicles are an important mechanism by which stem/progenitor cells might repair kidney injury. Here, this review will discuss the latest advances concerning the application potential of stem/progenitor cell-derived extracellular vesicles in renal diseases, including the aspects as follows: anti-inflammatory, proliferation-promoting and anti-apoptotic, proangiogenic, antifibrotic and renal cancer progression-promoting. Therefore, stem/progenitor cell-derived extracellular vesicles may be a promising treatment tool for renal diseases.

## Background

Acute kidney injury (AKI) is associated with high mortality, prolonged hospitalization, and substantial health care resource consumption and can even lead to progressive chronic kidney disease (CKD), including chronic kidney failure [1]. The overall AKI incidence rate among adults and children was 23.2%, with the highest incidence rate of 31.7% occurring in the critical care setting. The severe AKI incidence rate was 2.3% [2]. In recent years, although the treatment of AKI and CKD has achieved good results, there is a need to develop clinical effective and novel treatments for the patients. In both human and animal models, the use of stem/progenitor cells such as bone marrow mesenchymal stromal cells (BMMSC) has been shown to promote the recovery of kidney diseases such as AKI and CKD [3, 4]. There are a lot of controversies about the mechanisms by which this occurs, with two mechanisms prevailing: the first is that stem/progenitor cells home to the injured kidney and engraft and differentiate into functional cells, replacing injured cells, a so-called “cellular pathway”, and the second is that stem/progenitor cells secrete humoral factors to promote regeneration, thereby improving kidney injury, a so-called “paracrine pathway” [5]. Up to now, the research results are more inclined to the latter, i.e., stem/progenitor cells accomplish the therapeutic effect through secretion function rather than the differentiation function [6, 7]. Stem cell therapy was observed to have beneficial renal protection under a variety of kidney pathologies, which was only a result of stem cell transdifferentiation or fusion, yet differentiation of stem cells into enough number of cells to reconstitute renal parenchymal organs was undetected [8–12]. Therefore, stem cell implantation and differentiation are unlikely to occur, and its role in the repair of renal tissue is minimal [5].

The hypothesis that stem cells may exert their effects via releasing active factors seems reasonable, because stem cells release soluble factors such as growth factors, cytokines, chemokines, and bioactive lipids, and these substances can bring about a wide range of physiological effects [13]. This hypothesis was first validated in mesenchymal stromal cells (MSCs), because MSC is the most widely studied stem cell type for treatment purpose. For example, MSC supernatant was found to independently promote renal tubular cell survival [9]. Recently, stem cells were found to release both soluble factors and specific extracellular vesicles (EVs). In 2009, mesenchymal stromal cell-derived EVs (MSC-EVs) were reported to protect the kidney from acute tubular injury [14]. Therefore, stem/progenitor cell-derived EVs (SC-EVs) is an important mechanism by which stem cells might repair kidney injury.

Here, we briefly review the biological characteristics of EVs and the action mechanism of SC-EVs on target cells and detailedly discuss the application potential of SC-EVs in the treatment of renal diseases.

## Biological characteristics of EVs

Almost all cell types can secrete EVs containing cargoes (including proteins, lipids, and nucleic acids), and the EVs enter a variety of body fluids, such as blood, celiac fluid, pleural fluid, and articular fluid. The important functions of EVs include transferring signaling molecules to the distant cells via paracrine way to mediate intercellular communication, thereby participating in a variety of physiological and pathological processes (such as immune response, phenotype regulation, and angiogenesis). Therefore, EVs are considered to be important information carriers for regulating both gene expression and the phenotype of either adjacent or distal recipient cells through paracrine mechanisms [15], which in fact is also one of the important mechanisms underlying tissue/organ repair.

EVs are found to mediate intercellular communication between stem cells and the injured cells. On the one hand, SC-EVs can act on the injured cells. For example, the mouse embryonic stem cell-derived EVs (ESC-EVs) can reprogram the hematopoietic progenitor cells through delivering both proteins and mRNA coding for several pluripotent transcription factors; however, RNase was found to inhibit the biological effects of ESC-EVs, leading to the failure of ESC-EVs to reprogram hematopoietic cells [16]. Additionally, the endothelial progenitor cell-derived EVs (EPC-EVs) can activate the angiogenesis program via transferring mRNA contained in the EVs (such as miR-126 and miR-296) [17, 18]. These studies have identified that SC-EVs can alter gene expression in target cells via transferring proteins and nucleic acids. On the other hand, studies show that the injured cells secrete EVs that regulate the function of stem cells [8, 9, 19]. The injured cell-derived EVs can transfer specific signals to stem cells and trigger stem cell differentiation, which might be another underlying mechanism for the repair of pathological tissues. The stem cells and injured cells act on each other through EVs released by themselves to achieve bidirectional intercellular communication [20]. Therefore, the bidirectional intercellular communication of EVs may be an important mechanism for tissue/organ repair. In this article, we detailedly discuss the action of SC-EVs on the target cells in kidney.

## Action mechanism of SC-EVs on target cells

### Immune regulatory activity of SC-EVs

A variety of SC-EVs (such as embryonic stem cell-derived EVs, inducing pluripotent stem cell-derived EVs, adipose stem cell-derived EVs, neural stem cell-derived EVs) can mimic stem cells to exert immune regulatory activity [21–25]. For example, MSC-EVs were reported to inhibit the expression of IFN-γ in peripheral blood mononuclear cells in patients with type 1 diabetes mellitus, increase the production of anti-inflammatory factors (such as TGF-β, IL-10), decrease the number of Th17 cells, increase the regulatory T cells (Treg) count, and convert IFN-γ-producing T cells into anti-inflammatory T helper 2 (Th2), and inhibit the proliferation of B cells [26–28]. In addition, the exogenous SC-EVs were observed to affect the repair function of endogenous stem cells by regulating the inflammatory microenvironment. For example, in vitro experiments have shown that MSC-EVs can convert macrophages condition from M1 to M2 at the cellular level, then M2 macrophage-derived EVs could promote Treg cells’ formation, thereby establishing an anti-inflammatory microenvironment suitable for endogenous stem cells’ function [29]. Therefore, the evidences from the above studies can serve as basis for the application of SC-EVs in kidney diseases.

### Regulatory effect of SC-EVs on cell phenotype

SC-EVs have also been identified to exert regulatory effect on the phenotype of target cells through transferring nucleic acid substances (such as mRNA, miRNA) to the target cells [16–18, 30–33]. For example, by high-throughput RNA sequencing and functional analysis, it was demonstrated that the high expression of miR-145 in the umbilical cord MSC-EVs reduced scar formation due to inhibition of fibroblast proliferation via inhibiting TGF-β2/SMAD2 pathway [34]. Regulatory effect of SC-EVs on cell phenotype might be another underlying mechanism for the repair of kidney tissues, and we will detailedly discuss the application potential of SC-EVs in renal diseases via the effect as follows.

## Therapeutic potentials of SC-EVs for kidney diseases

**Fig. 1:**
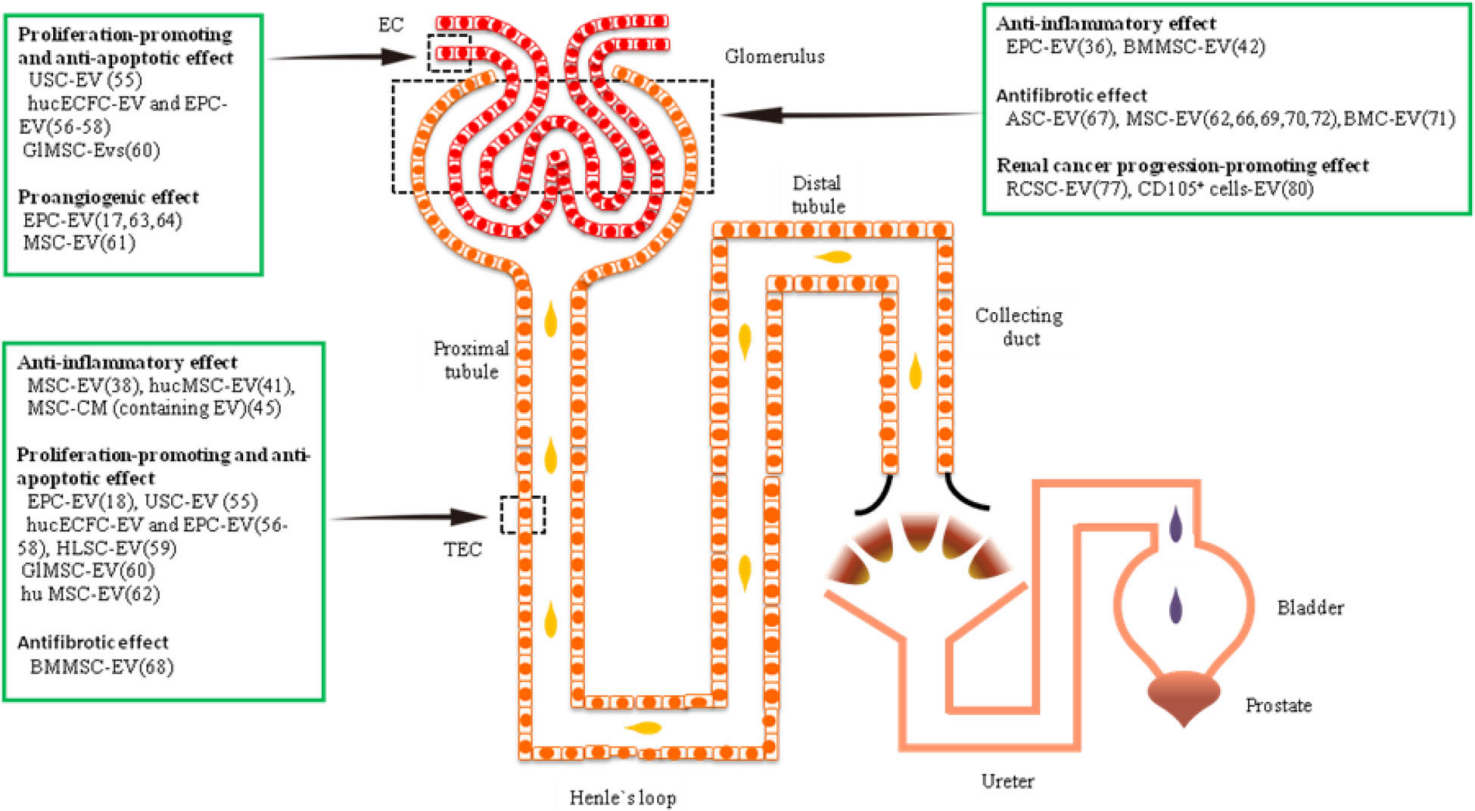
Effects of stem/progenitor cell-derived extracellular vesicles in renal diseases. The stem/progenitor cell-derived extracellular vesicles exert a series of renoprotective and regenerative effects on the injured tissues through different paracrine mechanisms in renal diseases, but they have also been shown to exert harmful effect on certain renal diseases or even aggravate disease progression. The effects include the aspects as follows: anti-inflammatory, proliferation-promoting and anti-apoptotic, proangiogenic, antifibrotic, and renal cancer progression-promoting. EV, extracellular vesicles; EC, endothelial cells; TEC, tubular epithelial cells; USC, urine-derived stem cells; hucECFC, human umbilical cord blood-derived endothelial cell colony-forming cells; EPC, endothelial progenitor cells; MSC, mesenchymal stromal cells; GlMSC, MSCs derived from the glomeruli; hucMSC, human umbilical cord MSCs; MSC-CM, mesenchymal stem cell-derived conditioned medium; HLSC-EV, human liver stem cell-derived extracellular vesicles; huMSC, human adult mesenchymal stem cells; BMMSC, bone marrow-derived mesenchymal stem cells; ASC, adipose stem cells; BMC, bone marrow cells; RCSC, renal cancer stem cells

The SC-EVs are delivered to the kidney tissues via intraperitoneal, arterial, venous, intrathecal, intraosseous, or renal subcapsular injections. They exert a series of renoprotective and regenerative effects on the injured tissues through different paracrine mechanisms, including immunosuppression and immunomodulation, promotion on proliferation and differentiation, anti-apoptosis, proangiogenesis, and anti-fibrosis [35]. In spite of the beneficial effect of SC-EVs on kidney diseases, endogenous SC-EVs have also been shown to exert harmful effect on certain renal diseases or even aggravate disease progression, for example, renal cancer stem cell-derived EVs (RCSC-EVs) have been found to promote tumor growth and metastasis. Collectively, we summarize the latest studies on therapeutic potentials of SC-EVs for kidney diseases **(**Fig. 1, Table 1).

**Table 1:** The application potential of stem/progenitor cell-derived extracellular vesicles in kidney diseases

Stem cell type releasing EVs	Animal models	Transferring materials	Target cells	Biogenesis mechanisms	Biological effects	References
EPC	Anti-Thy1.1glomerulonephritis rat model	miRNA	Injured glomerular cells	Inhibit leukocyte infiltration and mesangial cell activation	Improve kidney function	[36]
	IRI rat model	miRNA (miR-126 and miR-296)	Hypoxic renal resident cells	Alter the proliferative phenotype of hypoxic renal resident cells and promote angiogenesis	Protect the kidney from ischemic acute injury	[18, 51]
	SCID mice	mRNA associated with NOS and PI3K/AKT	ECs	Activate the PI3K/AKT signaling pathway	Trigger neovascularization, promote angiogenesis	[17]
BMMSC	IRI rat model	miR-218	Injured ECs	Enhance endothelial cell migration and stimulate a reparative phenotype	Treat microvascular endothelial injury	[64]
	IRI rat model	Chemokine receptors and complement-related proteins	Macrophage and apoptotic cells	Inhibit macrophage activity and promote phagocytosis of apoptotic cells	Prevent early renal injury	[37]
	Rat renal transplant model for acute rejection	EVs	**–**	Induce accumulation of T cells and B cells in renal tissue	Immunomodulatory of the immune system	[41]
	IRI rat model	Adhesion molecules, mRNA and miRNA	TECs	Reduce TECs apoptosis and increase TECs proliferation	Protect from AKI and from subsequent chronic renal damage	[62]
	Type 2 diabetic mice and insulin-resistant diabetic mice model	EVs	TECs	Suppress the EMT of TECs	Attenuated renal fibrosis	[68]
	UUO mouse model	miRNA	Proximal TECs	Enhanced inhibition of TGF-β1-induced EMT	Improve renal function	[66]
	UUO mouse model	miRNA-let7c	Damaged kidney cells	Reduce collagen accumulation and fibrotic-related gene expression	Alleviate kidney fibrosis	[80]
hWJMSC	IRI rat model	miRNA	ECs	Inhibit the expression of CX3CL1 and reduce the quantity of CD68^+^macrophages	Ameliorate renal injury in both the acute and chronic stage	[43]
USC	Type I diabetic rat model	Growth factors, TGF-β1, angiopoietin, and BMP-7	**–**	Inhibit podocyte apoptosis and promote vascular regeneration and cell survival	Prevent kidney injury from diabetes	[55]
ECFC	IRI rat model	miR-486-5p	ECs	Target at PTEN/Akt pathway	Protect the kidney from IRI injury	[56–58]
HLSC	SCID mouse model of AKI	EVs	Tubular cell	Stimulate proliferation and inhibit cell apoptosis	Promote AKI recovery	[59]
GlMSC	IRI rat model	miRNAs	TECs	Activate TEC proliferation	Promote the recovery of AKI	[60]
RCSC	**–**	HLA-G, costimulatory molecules and adhesion molecules	DCs	Suppress immune response	Tumor immune escape and immune tolerance	[76]
	**–**	Genes associated with matrix remodeling, cell migration, tumor growth, and angiogenesis	MSCs	Induce a pro-tumorigenic phenotype	Promote tumor growth	[77]
RCSC(CD105^+^ cells)	SCID mice	Proangiogenic mRNA and miRNA	Epithelial cells and ECs	Trigger angiogenesis and promote the formation of premetastatic niche	Promote renal cancer progression and lung metastases	[79]

*EVs* extracellular vesicles, *EPC* endothelial progenitor cells, *MSC* mesenchymal stromal cells, *BMMSC* bone marrow-derived mesenchymal stem cells, *hWJMSC* human Wharton-Jelly MSCs, *USC* urine-derived stem cells, *ECFC* endothelial colony-forming cells, *HLSC* human liver stem cells, *GlMSC* MSC-derived from the glomeruli, *RCSC* renal cancer stem cells, *IRI* ischemia-reperfusion injury, *SCID* severe combined immunodeficient, *UUO* unilateral ureteral obstruction, *AKI* acute kidney injury, *NOS* nitric oxide synthase, *BMP-7* bone morphogenetic protein-7, *ECs* endothelial cells, *TECs* tubular epithelial cells, *DCs* dendritic cells, *EMT* epithelial–mesenchymal transition

### Anti-inflammatory effects

On early AKI stage, SC-EVs have shown potent anti-inflammatory potentials in rodent kidney disease models. For example, in experimental anti-Thy1.1 glomerulonephritis, EPC-EVs were found to localize within injured glomeruli, and further studies have shown that EPC-EVs treatment protected the podocyte marker synaptophysin and the endothelial cell antigen (RECA-1) and inhibited Thy1.1 antibody/complement-induced cell apoptosis and the deposition of C5b-9/C3 in mesangial cell, thereby protecting renal function (Fig. 1, Table 1) [36]. Additionally, in ischemia reperfusion-induced AKI mouse model, C-C motif chemokine receptor 2 (CCR2) enriched in MSC-EVs was found to inhibit CCL2-mediated macrophage activity and the complement-related proteins (CD59, C5, C3, and C4A) released by MSC-EVs were found to contribute to the phagocytosis of apoptotic cells and protection against early renal injury (Table 1) [37].

On advanced AKI stage, the molecules released by SC-EVs have been found to promote renal tissue repair through acquired immune response [38, 39]. For example, in cisplatin-induced AKI mouse model, human umbilical cord MSC-derived EVs (hucMSC-EVs) were found to upregulate autophagy-related gene (ATG5/ATG7) expression in renal TEC, reduce the production of inflammatory factor TNF-α and IL1-β, and increase the number of renal tubular anti-apoptotic protein, thereby attenuating renal injury (Fig. 1) [40]. Additionally, in a rat renal transplant model for acute rejection, BMMSC-EVs were found to induce accumulation of T cells and B cells in renal tissues, decrease the number of NK cells, and decrease TNF-α expression (Fig. 1, Table 1) [41].

It is worth noting that there are also reports about the harmful effect of EV-derived cytokines on renal repair. On early AKI stage, the bioactive substances (cytokines, growth factors, and lipid mediators) released by EVs were found to increase apoptosis of tubular epithelial cells and endothelial injury, thus worsening tissue damage through activation and recruitment of neutrophils, M1 type macrophages, and other lymphocytes [39]. For example, in the toxicant-induced AKI model, the use of BM-MSC was found to result in the increase of a large number of granulocytes and aggravation of renal injury [42].

Besides on AKI, large amounts of data have also shown the biological effects of SC-EVs on CKD in both humans and animal models. CX3CL1 chemokine is the ligand of CX3CL1 receptor on macrophages and T cells. Studies have shown the reduced expression of CX3CL1 in AKI rats and the attenuation of AKI induced by the neutralization effect of CX3CL1 (Table 1) [43, 44]. It is worth noting that long-term administration of human MSC-conditioned medium (containing EVs) in a rat model of established CKD is associated with increased expression of CX3CL1 in TEC, indicating its beneficial effect on TEC repair [45]. Moreover, studies on CKD patients have demonstrated the significant therapeutic effect of MSC-EV treatment evidenced by significant improvement in a series of evaluation indicators (such as glomerular filtration rate, urinary albumin to creatinine ratio, serum uric acid, and serum creatinine levels); the analysis on the CKD patients’ renal pathology showed an increase in the number of renal progenitor cells (i.e., CD133/Ki-67 renal tubular cells) in the MSC-EV treatment group as compared with the control group, indicating that the regeneration process of progenitor cells in the injured kidney has been initiated by MSC-EVs [46].

### Proliferation-promoting and anti-apoptotic effects

Several types of renal injury are all characterized by renal TEC damage and dysfunction and loss of endothelial cells [47, 48]. Therefore, the functional recovery of renal TEC and vascular endothelial cells is crucial for the repair of renal injury. Several studies have shown that EVs released by exogenous stem cells/precursor cells and renal resident cells exert repair activity on toxic or ischemic kidney injury [49, 50].

EPC-EVs protected against progression of renal ischemia-reperfusion injury into CKD by inhibiting capillary rarefaction and glomerulosclerosis [18]. The protective effect of EPC-EVs is achieved mainly through transfer of angiogenic miRNA (miR-126 and miR-296) to hypoxic renal resident cells and alteration of their proliferative phenotype (Fig. 1, Table 1) [18, 51]. EPC-EVs were found to affect endothelial cells as well as renal tubular cells [52, 53]. For example, in AKI rat model, labeled EPC-EVs were found in tubular epithelial cells (TEC) after tail vein injection, which also exhibited proliferation-promoting activity in vitro [18]. Moreover, in type I diabetic rat model, urine stem cell-derived EVs (USC-EVs) were shown to protect podocytes and inhibit apoptosis of renal TEC, inhibit the overexpression of Caspase-3, and promote the proliferation of glomerular endothelial cells (ECs). The growth factors, TGF-β1, angiopoietin, and bone morphogenetic protein-7 contained in USC-EVs, were shown to promote angiogenesis and cell survival (Fig. 1, Table 1) [54, 55].

Studies on ischemic AKI mouse model have demonstrated that intravenous injection of EVs of human umbilical cord blood-derived endothelial cell colony-forming cells and highly proliferative and angiogenic EPC could attenuated renal injury through improvement in the necrosis and of apoptosis of renal TEC; moreover, these EVs containing miR-486-5p were found to target at PTEN/Akt pathway through transferring miRNA to ECs, leading to reduction in renal PTEN level and activation of Akt, thereby protecting kidney against ischemia reperfusion injury (IRI) (Fig. 1, Table 1) [56–58]. Additionally, studies have confirmed the therapeutic efficacy of other stem cell (e.g., MSC, human liver stem cells)-derived EVs for AKI and other kidney injury (Fig. 1, Table 1) [59].

Besides exogenous SC-EVs, endogenous renal stem/progenitor cell-derived EVs also have beneficial effects in repairing kidney damage. For example, MSCs derived from the glomeruli (GlMSC)-EVs were noted to improve renal function and attenuate ischemic injury by activating TEC proliferation, and this effect was mediated by miRNAs via GlMSC-EV transferring, and these miRNAs are involved in a variety of biological processes including cell communication, nucleic acid metabolism, and regulation of gene expression, triggering pro-regenerative process of TEC (Fig. 1, Table 1) [60]. This study further confirms the previous conclusion that nephrogenic MSC-EVs as carriers of proangiogenic signals contribute to recovery from AKI following IRI [61].

In conclusion, EVs from renal resident cells and exogenous stem/progenitor cells contribute to kidney repair after toxic or ischemic renal injury, whereas there are differences between the two types of EVs in biological activities. For example, the renoprotective effects of MSC-EVs released by renal resident cells were noted to be lower than that of exogenous MSC-EVs. Moreover, in IRI-induced AKI rat model, the renoprotective effect of renal tubular CD133^+^cell-derived EVs (T-CD133^+^-EVs) was significantly lower than that of GlMSC-EVs [60], and human MSC-EVs via intravenous injection were noted to reduce TEC apoptosis and increase TEC proliferation, but no similar biological effect was noted for fibroblast-derived EVs (Fig. 1, Table 1) [62]. Therefore, the different biological activities of these EVs remain to be studied further.

### Proangiogenic effects

The neovascularization plays an important role in kidney repair, with renal ECs playing a key role in this process. Numerous data have shown that EVs could initiate reprogram, induce angiogenesis phenotype formation, and enhance migration ability, thus promoting neovascularization by transferring its cargoes (e.g., bioactive molecules, specific mRNA, and miRNA) into ECs. For example, in patients with AKI associated with sepsis, intravenously injected EPC-EVs could reach into ECs and TEC, with posing direct effect on TECs in the presence of hypoxia (Fig. 1) [63]. Additionally, EVs of human vascular progenitor cells derived from renal arteries express endothelial-like phenotype, and in hypoxia-induced renal microvascular network injury model, high level of miR-218 transferred by these EVs was shown to enhance the migration of ECs after IRI (Fig. 1, Table 1) [64]. The miRNAs released by EPC-EVs (such as miR-126 and miR-296) have been shown to promote angiogenesis by reprogramming renal resident cells [18]. In addition, subcutaneous injection of EPC-EVs and matrigel matrix in mice with severe combined immunodeficiency disease was shown to transfer mRNA associated with nitric oxide synthase (NOS) and the PI3K/AKT signaling pathway to ECs, triggering neovascularization in human tubular vascular endothelial cells (Fig. 1, Table 1) [17]. After co-culturing with injured ECs, EVs released by human renal artery progenitor cells after undergoing radical nephrectomy were shown to enhance the migration ability of the injured ECs [64]. Therefore, these studies have demonstrated the feasibility of applying EVs derived from angiogenic progenitor cells for treatment of microvascular endothelial injury.

### Antifibrotic effects

EVs have been shown to exert protective effect in fibrotic pathological process in chronic renal inflammation [65, 66]. Many SC-EVs (such as adipose stem cell-derived EVs, MSC-EVs) were found to have anti-fibrotic effects, leading to attenuated renal fibrosis (Fig. 1, Table 1) [67, 68]. It is worth noting that upregulation of miRNA in extracellular vesicles by erythropoietin (EPO) may contribute to their enhanced renal protective effect [66]. For example, in UUO-induced renal tubular interstitial fibrosis mouse model, EPO was shown to promote the release of miR-144 in bone marrow cell-EVs, and these miR-144 targeted to tPA gene and then inhibited tPA/MMP9-mediated proteolysis network and blocked entrance of MMP9 into the mouse kidney so as to exert the protective effects of bone marrow cell-EVs on renal tubule basementmembrane [69, 70]. Additionally, in ischemia reperfusion-induced chronic kidney injury model, MSC-EVs were shown to reduce renal fibrosis (Fig. 1) [62, 71]. For example, in CKD rat model, EPO-pretreated MSC-EVs were shown to alleviate TGF-β1-induced fibrosis [66]. In the same model, EPO-treated EVs were enriched with gene-targeting miRNAs (miR-133b-3p, miR-294, miR-299, miR-499, miR-302, and miR-200), and these miRNAs have been shown to involve in mitochondrial apoptosis, oxidative stress pathways, and TGF-β and E cadherin pathway of mediating the epithelial-mesenchymal transition [66, 72]. In fact, MSC-EVs co-cultured with EPO were observed with greater anti-fibrotic effects both in vivo and in vitro in UUO model than that cultured alone (Fig. 1, Table 1) [66]. Therefore, EPO-treated SC-EVs may be important tools to exert protective effect in fibrotic pathological process via antifibrotic effects.

### Renal cancer progression-promoting effects

The tumor microenvironment is a complex cellular environment in which the tumor exists along with immune cells, fibroblasts, signaling molecules, and the extracellular matrix. Positive cell communication occurs between cancer cells and the adjacent cells (including infiltrating immune cells and stromal cells) in the microenvironment, and this intercellular communication is associated with multiple pathological processes including tumor establishment, progression, metastasis, and recurrence, thereby either promoting or inhibiting the pathological process of tumor [73]. EVs are considered to involve in signal transduction process between cancer cells and the adjacent cells [74]. Moreover, EVs are considered to be a novel complex mechanism of cell communication within the tumor microenvironment, and EVs released from tumor stem cells have been shown to achieve information communication through activating immune tolerance, cell proliferation, angiogenesis, metastasis formation, or other means [75].

In renal cancer, renal cancer stem cell-derived EVs have shown to inhibit the differentiation process of dendritic cells (DCs) from monocytes through significantly attenuating the expression of HLA-G, costimulatory molecules, and adhesion molecules that lead to immune suppression, favoring tumor immune escape that leads to immune tolerance (Fig. 1, Table 1) [76]. The EVs derived from cancer cells were shown to alter the function of non-immune cells in tumor stroma. For example, EVs derived from renal cancer stem cells were shown to induce a pro-tumorigenic phenotype on the surface of MSCs in microenvironment and increase expression of genes associated with matrix remodeling (COL4A3), cell migration (CXCR4, CXCR7), tumor growth (IL-8, osteopontin and myeloperoxidase), and angiogenesis. Importantly, EV-stimulated MSCs showed an enhanced capacity to induce the migration of renal tumor cells and vessel-like formation in vitro and supported tumor development and vascularization in vivo (Fig. 1, Table 1) [77]. Additionally, tumor-initiating stem cell population expressing MSCs’ marker (CD105) was present in human renal carcinomas, and these CD105^+^ cells revealed the properties of stem cells [78]. The EVs derived from CD105^+^ cells contain proangiogenic mRNAs and miRNAs that can trigger angiogenesis and formation of premetastatic niche, change the microenvironment for cancer development, and promote renal cancer progression and lung metastases (Fig. 1, Table 1) [79]. Therefore, cancer stem cell-derived EVs by engineering may be treatment targets for renal cancer in further studies.

## Prospection

SC-EVs with multiple features are a cellular product from stem cells that make them suitable for clinical therapies in renal diseases. SC-EVs may have many advantages. For example, compared with stem cells, SC-EVs possess the characteristic of lower immunogenicity because they lack the surface antigens on the surface membrane of stem cells. Thus, the injured recipient may accept EVs from different types of stem cells because of low immunogenicity. Moreover, the administration of SC-EVs instead of stem cells might also reduce some of the risks associated with cellular therapy (e.g., cytokine release syndrome, graft-versus-host disease). Thus, the potential advantages of stem cell-derived EV-based applications versus whole stem cell-based applications provide a rationale to develop an EV-based therapy for renal diseases.

To sum up, these above studies have demonstrated the feasibility of applying SC-EVs for kidney diseases. However, there are many challenges for the use of SC-EVs for clinical treatment in renal diseases. Several key issues pertaining to the use of EVs in clinical require further exploration. First, how to keep the activity of SC-EVs in vitro before engraftment in the renal parenchyma? Are cryopreserved SC-EVs as effective as fresh isolated SC-EVs? Second, how to meet the large-scale clinical production requirement of a sufficient quantity of SC-EVs? Third, how to select the approach of SC-EV transplantation into the renal parenchyma from intravenous or intrathecal or intraosseous or local injection? Fourth, how to track SC-EVs in the kidney? Thus, the development of specific tracking tools is required for further detecting the occurrence of SC-EVs. These questions will have to be answered before the widespread application of SC-EV therapy in clinical practice. Overall, the potential of stem cell-derived EVs in applications for renal diseases is highly promising.

## Abbreviations

AKI: Acute kidney injury
ASC: Adipose stem cells
BMC: Bone marrow cells
BMMSC: Bone marrow-derived mesenchymal stromal cells
BMP-7: Bone morphogenetic protein-7
CKD: Chronic kidney disease
DCs: Dendritic cells
ECFC: Endothelial colony forming cells
ECs: Endothelial cells
EMT: Epithelial–mesenchymal transition
EPC: Endothelial progenitor cells
EVs: Extracellular vesicles
GlMSC: MSCs derived from the glomeruli
HLSC-EV: Human liver stem cell-derived extracellular vesicles
hucECFC: Human umbilical cord blood-derived endothelial cell colony forming cells
hucMSC: Human umbilical cord MSCs
huMSC: Human adult mesenchymal stem cells
hWJMSC: Human Wharton-Jelly MSCs
IRI: Ischemia-reperfusion injury
MSC-CM: Mesenchymal stem cell-derived conditioned medium
NOS: Nitric oxide synthase
RCSC: Renal cancer stem cells
SCID: Severe combined immunodeficient
TEC: Tubular epithelial cells
USC: Urine-derived stem cells
UUO: Unilateral ureteral obstruction
